# The structural impact of cancer-associated missense mutations in oncogenes and tumor suppressors

**DOI:** 10.1186/1476-4598-10-54

**Published:** 2011-05-16

**Authors:** Henning Stehr, Seon-Hi J Jang, José M Duarte, Christoph Wierling, Hans Lehrach, Michael Lappe, Bodo MH Lange

**Affiliations:** 1 Max-Planck Institute for Molecular Genetics, Structural Proteomics/Bioinformatics Group, Otto-Warburg Laboratory, Boltzmannstrasse 12, 14195 Berlin, Germany; 2 Freie Universität Berlin, Department of Biology, Chemistry and Pharmacy, Takustrasse 3, 14195 Berlin, Germany; 3 Max-Planck Institute for Molecular Genetics, Department of Vertebrate Genomics, Ihnestrasse 73, 14195 Berlin, Germany; 4 Paul Scherrer Institut, Biomolecular Research, Biology and Chemistry, 5232 Villigen PSI, Switzerland

## Abstract

**Background:**

Current large-scale cancer sequencing projects have identified large numbers of somatic mutations covering an increasing number of different cancer tissues and patients. However, the characterization of these mutations at the structural and functional level remains a challenge.

**Results:**

We present results from an analysis of the structural impact of frequent missense cancer mutations using an automated method. We find that inactivation of tumor suppressors in cancer correlates frequently with destabilizing mutations preferably in the core of the protein, while enhanced activity of oncogenes is often linked to specific mutations at functional sites. Furthermore, our results show that this alteration of oncogenic activity is often associated with mutations at ATP or GTP binding sites.

**Conclusions:**

With our findings we can confirm and statistically validate the hypotheses for the gain-of-function and loss-of-function mechanisms of oncogenes and tumor suppressors, respectively. We show that the distinct mutational patterns can potentially be used to pre-classify newly identified cancer-associated genes with yet unknown function.

## Background

Cancer genomics studies aim to provide new insights into the molecular mechanisms that lead to tumorigenesis. To this end, second generation sequencing facilitated the extensive analysis of the genome and kinome landscapes of diverse cancer types [1-7]. These approaches provide detailed information on the frequency and position of single point mutations as well as structural aberrations of cancer genomes such as small insertions and deletions, focal copy number alterations, and genomic rearrangements. The findings show that the complexity of each cancer genome is far greater than expected and that extensive variations exist between different cancer types as well as between different tumor samples of the same cancer type. While additional sequencing data is continuously generated, the identified mutations can currently not be functionally characterized in an automated way that keeps pace with the output and development of new sequencing technologies.

Here we present an analysis of the structural consequences caused by missense mutations that occur in the most frequently mutated genes in eight common cancer types. Our analysis focuses on four structural features: solvent accessibility, protein stability, proximity to functional sites and spatial clustering. We assess the effects of ~2000 cancer-associated mutations in oncogenes and tumor suppressors and compare them to the effects of natural variants and randomized mutations.

Several previous studies have analyzed properties of cancer mutations based on features that can be derived from sequence data. Such properties include sequence conservation of mutated positions [8], ancestral alleles and substitution propensities [9], and analysis of domain types targeted by mutations [10]. In our analysis, we focus on mechanisms of cancer mutations that have a consequence at the structural level. This makes our method complementary to sequence-based approaches. Another significant body of work has been published on consequences of mutations in a structural context [11-15]. These studies differ in that either they focus on estimating the effects of individual mutations or they use different sets of disease mutations. We will show that by breaking the set of cancer mutations into more specific subclasses, functionally relevant information is revealed that may be missed otherwise. In particular, we find distinct mutational patterns in oncogenes and tumor suppressors reflecting mechanisms of functional activation and inactivation, respectively. We statistically validate the observations and show to what extend these differences can be used for predictive purposes.

## Methods

### Cancer mutation dataset (*Mut*)

Somatic mutations for eight cancer types (breast, prostate, stomach, colon, pancreas, thyroid, kidney, lung) were taken from the COSMIC database (Catalogue of Somatic Mutations in Cancer) [16]. For these cancer types, all genes were extracted from COSMIC (v49) for which crystal structures (each of length ≥30aa and together covering at least 25% of the gene) were available and which were part of the "Cancer Gene Census" category of COSMIC. For genes in this category a comprehensive literature screening has been conducted. A cut-off of 6 distinct missense mutations for each gene in the structurally resolved regions was chosen based on the observation that genes with very few mutations show high statistical fluctuations. As we exclusively consider missense mutations, we refer to them as "mutations" hereafter. The genes and the corresponding mutations were subsequently separated into the two datasets *Onc *and *Sup *representing the subset of mutations in oncogenes and tumor suppressors, respectively (see Table 1). A graphical overview of the mutations along the sequence as well as the coverage of the crystal structures is provided in Additional File 1, Figure S1. The set of genes results from the described automatic selection procedure without any manual intervention.

**Table 1:** Overview of genes

Gene name	Length (AA)	Mut	SNP	PDB codes (sequence range)
*Oncogenes*				

*AKT1*	478	6	5	1UNQA (1-123), 3CQWA (144-480)
*BRAF*	766	46	3	3D4QA (433-726), 3NY5A (153-237)
*EGFR*	1210	224	9	1YY9A (25-642), 1XKKA (695-1022)
*GNAS*	394	12	9	1AZSC (1-394)
*HRAS*	189	19	0	4Q21A (1-189)
*KIT*	976	9	9	2EC8A (1-519), 3G0EA (544-935)
*KRAS*	188	85	1	3GFTA (1-164)
*MET*	1408	24	30	2UZXB (25-740), 3DKCA (1049-1360)
*NRAS*	189	9	1	3CONA (1-172)
*PIK3CA*	1068	148	17	2RD0A (1-1068)
*PTPN11*	593	7	6	2SHPA (3-529)
*RET*	1114	24	3	2IVSA (705-1013), 2X2UA (29-270)

*Tumor Suppressor Genes*				

*CDH1*	882	17	3	2O72A (155-367)
*CDKN2A*	156	76	10	1BI7B (1-156)
*FBXW7*	707	34	4	2OVRB (263-707)
*MLH1*	756	8	3	3NA3A (1-347)
*MSH2*	934	12	17	2O8BA (1-934)
*PTEN*	403	93	2	1D5RA (8-353)
*RB1*	928	7	9	2R7GA (380-787), 2QDJA (52-355), 2AZEC (829-874)
*SMAD4*	552	51	3	1DD1A (285-552)
*STK11*	433	30	1	2WTKC (43-347)
*TP53*	393	826	17	2VUKA (94-312), 1AIEA (326-356)
*VHL*	213	216	16	1LM8V (54-213)
*WT1*	449	9	3	2PRTA (318-438)

Abbreviations: AA, amino acid, Mut, number of mutations, SNP, number of SNPs, PDB, Protein Data Bank

### Single nucleotide polymorphism dataset (*Snp*)

As a control set, we extracted single nucleotide polymorphism (SNP) data for the 24 genes from version 131 of the common variation database dbSNP [17]. Minor allele frequency data was only available for a small subset of dbSNP entries. Therefore, we excluded those SNPs that are annotated by dbSNP as disease-associated instead.

### Random control (*Rnd*)

As an additional control and as the null-model for the statistical analysis we generated random populations of mutations. These are sampled uniformly from the amino acid sequence. For each dataset (*Snp, Mut, Onc, Sup*), a random population is obtained by drawing 100 000 sets of mutations. Each such set has the same size as the corresponding set of observed mutations. Moreover, the number of mutations per structural domain is kept constant. This ensures that the geometry and sequence composition of the domains does not bias the results.

### Protein structures

Known crystal structures were taken from the Protein Data Bank [18]. The ones with the largest sequence coverage and with the best crystallographic resolution were chosen.

### Structure modeling

Structure models were built using an in-house pipeline based on established homology modeling principles. Templates were identified by a psi-blast search with 5 iterations [19]. Models were built using distance geometry and subsequent simulated annealing refinement.

### Statistical analysis - odds ratios

The structural features described below are evaluated for each gene in terms of the odds ratio of observed over expected behavior. Expected values are calculated by generating a large population of randomized sets of mutations (as described above) and evaluating the property (e.g. fraction of solvent accessible residues in the structure) averaged over the population.

### Structural feature - solvent accessibility

Solvent accessibilities were computed using the NACCESS software [20]. NACCESS calculates the relative solvent accessibility (RSA) using a water probe. Residues were considered to be solvent accessible or "surface residues" if the RSA was greater than 15%. The odds ratio is calculated as observed over expected fraction of surface mutations in a gene.

### Structural feature - protein stability

To estimate the effect of a mutation on protein stability we used version 3.0 beta of the FoldX software [21]. Calculations were performed using the BuildModel command with default parameters. Mutations are considered destabilizing if the difference in free energy between wild type and mutants (*ΔΔG*) exceeds 5 kcal/mol. This value is a typical lower bound for the stability of globular proteins [22]. Otherwise, the mutation is considered neutral. The odds ratio is calculated as observed over expected fraction of destabilizing mutations in a gene.

### Structural feature - proximity to functional sites

A mutation is considered proximal to a functional site if it occurs at or in contact with a functional residue where contact is defined as the C-beta atoms of the respective residues being no more than 8Å apart (C-alpha for glycine).

Functional site annotations were derived from public databases (UniProt release 2010_10 [23], Catalytic Site Atlas version 02.02.12 [24], PhosphoSitePlus as of 2010-10-15 [25]). We extracted the following categories of functional site annotations: Enzyme active sites, ATP/GTP binding sites, phosphorylation sites, ubiquitination and other post-translational modifications (acetylation, methylation, and glycosylation). The odds ratio is calculated as observed over expected fraction of mutations proximal to a functional site.

### Structural feature - spatial clustering

To measure whether a set of mutations is spatially clustered in the structure, we divide the protein into structurally defined domains and calculate a spatial clustering value *C *as follows:

where *d*_*i,j *_is the Euclidean distance in the structure between the side chain centroids of residues *i *and *j *and *N *is the number of such residue pairs. We used the C-alpha position for glycines and residues with unavailable side chain coordinates.

The domains are structurally defined using the DomainParser method [26]. Only distances within domains are evaluated. The subdivision into domains is crucial to avoid bias due to the size and domain architecture of the protein. The odds ratio is calculated as observed over expected clustering value of the mutations in a gene.

### Statistical analysis - p-values

The statistical significance of the observations was assessed by calculating the p-value under the null-model assumption of a uniform distribution of the mutations. In the cases with a binary outcome for each position (surface/core, neutral/destabilizing) the null-model distribution is binomial and the p-value can be calculated analytically. For spatial clustering and proximity to functional sites it has to be obtained from the random control population. Let f be such an empirical null-model distribution with mean *m*. Then, the p-value of an observation *O *is approximated as the fraction of individuals *v *in the population with f(*v*) ≥ f(*O*) if *O *≥ *m *or f(*v*) ≤ f(*O*) if *O *<*m*.

### Statistical analysis - jackknife test

**Figure 1:**
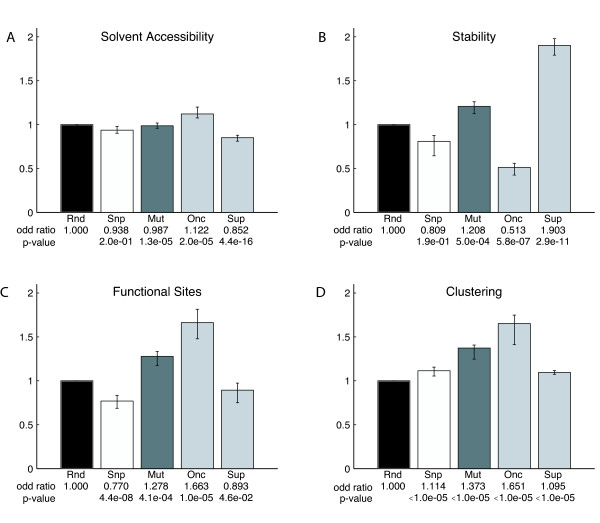
**Structural impact of mutations**. The columns show the structural properties of random mutations (*Rnd*), natural variations (*Snp*) and cancer mutations (*Mut*). Cancer mutations are further analyzed separately as mutations of oncogenes (*Onc*) and mutations of tumor suppressor genes (*Sup*). The error bars indicate the variability of the data under the jackknife test. The reported values are the odds ratios averaged over the genes in the dataset. The p-values are calculated over all mutations within a dataset. A, observed over expected fraction of mutations occurring at the protein surface. *Onc *show significantly more and *Sup *significantly less solvent accessible mutations. B, observed over expected fraction of destabilizing mutations. *Onc *mutations are less often destabilizing, while *Sup *mutations disrupt stability far more often than the controls. C, observed over expected functional site mutations. Functional sites are more frequently mutated in *Onc *than in *Sup*. D, observed over expected spatial clustering of mutations. Mutations particularly in *Onc *are significantly more clustered than expected by chance.

To assess the robustness of the data against outliers, we applied a jackknife test. This test is a bootstrapping procedure where the results are being recalculated multiple times, each time leaving out one gene from the original dataset. Taking the maximum and the minimum over this procedure for all genes yields an interval around the value of the original dataset. These intervals are shown as error bars in the Figure 1.

### Linear classifiers

Linear classifiers were automatically calculated using Fisher's linear discriminant method, which provides a good compromise between finding the optimal solution in the linearly separable case and being robust to outliers [27]. To test the robustness of the classification we applied a leave-one-out cross validation procedure. In each step, one gene is temporarily removed from the training set. The classifier is recalculated on the subset and we test whether it is able to correctly predict the class membership of the excluded gene.

### Additional material

Information on genes, mutations, SNPs and functional annotations that were used in the analysis is available in electronic form as Additional Tables S1-S4 (Additional Files 2, 3, 4, 5).

## Results

In this study we analyze the structural impact of a large number of cancer mutations in oncogenes and tumor suppressors. We evaluate the impact with respect to four structural features. We focused on eight selected tumor entities that are among the most frequent and lethal types. The *Mut *dataset extracted from the COSMIC database [16] comprises 1992 mutations in 24 cancer genes. This set contains many classical cancer genes that are involved in major signaling pathways (i.e. TGFb, EGFR, MAPK, PI3K/AKT signaling). The genes with their corresponding mutations were subdivided into the classes of tumor suppressor (*Sup*) and oncogenes (*Onc*) as shown in Table 1, representing two common mechanisms through which tumorigenesis is initiated: via gain-of-function of oncogenes and loss-of-function of tumor suppressors [28]. As a control, we use a set of 204 non-disease-related SNPs (the *Snp *dataset) extracted from NCBI's database dbSNP [29].

In the following we present the results for the four structural properties. In Figure 1 we report the average odds ratios over the genes in the respective set (*Snp, Mut, Onc, Sup*).

### Solvent accessibility

As the first property, we investigated whether mutations occur at the surface or in the core of the protein. Figure 1A shows that there is little difference between the SNPs (*Snp*, 0.938) and cancer mutations (*Mut*, 0.987). However, a separate analysis of oncogenes and tumor suppressors reveals that mutations in oncogenes occur significantly more often at the surface (1.122, p-value 2e-5), while mutations in tumor suppressors are overrepresented in the core (0.852, p-value 4.4e-16).

### Protein stability

We calculated the impact that the mutations of the different datasets have on protein stability. The calculations were performed with the FoldX software [21]. A recent assessment has shown that this method is currently among the best methods for calculating stability changes upon mutation [30]. The results of this analysis (Figure 1B) show a distinct difference between oncogenes and tumor suppressors. Tumor suppressors display a significant overrepresentation of mutations that destabilize the protein (1.903, p-value 2.9e-11) with an almost four-fold increase compared to oncogenes with significantly fewer destabilizing mutations (0.513, p-value 5.8e-7).

### Proximity to functional sites

Next we assessed whether the mutations in our dataset occur proximal to known functional sites and thus are likely to directly influence protein function. For this we extracted 258 annotated functional sites from public databases. The results are shown in Figure 1C. Cancer mutations in oncogenes (*Onc*) have a tendency to specifically target functional sites (1.663, p-value 1e-5), while in tumor suppressors (*Sup*) mutations proximal to functional sites are significantly underrepresented (0.893, p-value 4.6e-2). Functional site mutations are also significantly underrepresented in the *Snp *data set (0.770, p-value 4.4e-8).

**Figure 2:**
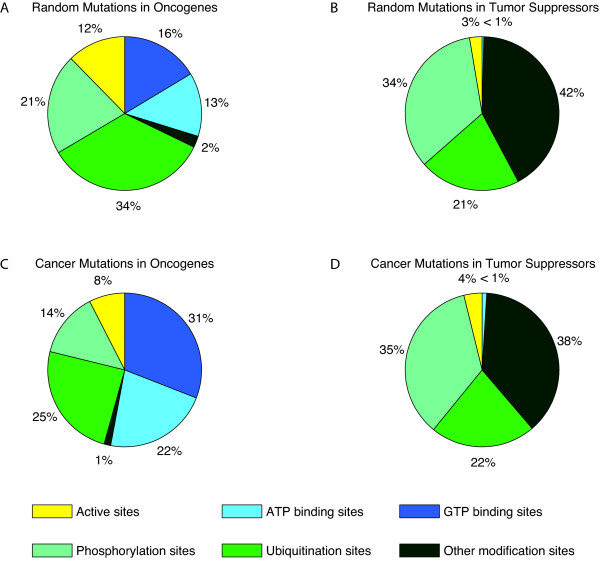
**Distribution of functional site mutations**. Distribution of mutations affecting functional sites in oncogenes (*Onc*) and tumor suppressors (*Sup*) compared to distribution of random mutations. A and B, distribution obtained by random sampling of positions in *Onc *and *Sup*, respectively. C, distribution of functional site mutations in *Onc*. ATP and GTP binding sites in *Onc *are significantly more often mutated than expected by chance. D, distribution of functional site mutations in *Sup*. Observed distribution does not differ significantly from expected random distribution.

Further, we investigated whether particular types of functional sites are more often mutated than expected. Figure 2 shows the observed distribution of functional site mutations in oncogenes and tumor suppressors compared to the distribution expected for randomized mutations. For oncogenes, ATP and GTP binding sites are significantly overrepresented among the mutated functional sites (31% compared to 16%, p-value 4.95e-11 (ATP) and 22% compared to 13%, p-value 4.86e-07 (GTP)). The results for tumor suppressors show no apparent differences between observed and random distribution.

### Spatial clustering

Next we wanted to test whether cancer mutations have a tendency to co-localize in spatial clusters. Figure 1D shows that cancer mutations in oncogenes are highly clustered (1.651), while tumor suppressor mutations behave similar to SNPs (1.095 compared to 1.114). Both are significantly more clustered than random (p-value <1e-5). The small error bar for *Sup *indicates that all tumor suppressors have similar clustering behavior.

In this case, the p-values result from the fact that a spatial clustering as high as the one for either of the sets *Snp, Mut, Onc *or *Sup *was never observed in the random reference population of size 100 000. Hence, the p-value is at most 1e-05.

### Redundancy in the dataset

The dataset contains three members of the RAS family, which exhibit high sequence similarity. This is a result of the automatic gene selection. To check for a possible bias introduced by this gene family we recalculated the average values with only one RAS gene and found that the conclusions are unchanged and are still supported by the significance values.

### Classification of cancer genes based on structural features

**Figure 3:**
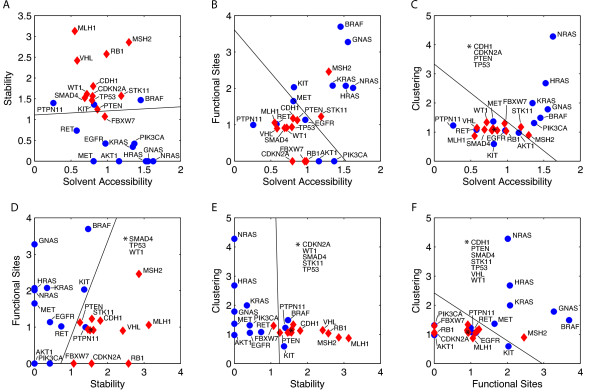
**Linear classification of cancer genes**. The different pairs of structural features are shown as scatter plots in A-F. Oncogenes are depicted as blue dots, tumor suppressors as red diamonds. The separating linear functions have been calculated using Fisher's linear discriminant method. The classifiers in A, D and E show the best training performance.

Given the distinct average behavior of the two cancer gene classes, we investigated to what extend this behavior is reflected at the individual gene level and to what extend it can be used for predictive purposes. To examine the discriminatory power of the structural features, the features were plotted in pairwise combinations (Figure 3). Each data point corresponds to one individual gene with oncogenes and tumor suppressors shown as blue dots and red diamonds, respectively. The values on the axes are the odds ratios for the feature values. We calculated linear classifiers trained on the two sets using Fisher's discriminant method [27].

Visually, the two classes are well-separated for feature combinations shown in Figure 3A, 3D and 3E. For combinations in Figure 3B, 3C and 3F the two subpopulations overlap more. Nevertheless, in all six plots there are areas exclusively populated by either class.

We have systematically evaluated the discriminatory power of the different feature combinations (see Table 2) by leave-one-out cross validation. We find that the combination of the two features *functional sites *and *stability *(Figure 3D) classifies best with a performance of 95.83%. The plots in Figure 3A and 3E (*stability *vs. *surface accessibility, clustering *vs. *stability*) display a cross validation performance of 83.33% and 79.17%, respectively. The other feature combinations possess modest classification power.

**Table 2:** Performance of linear classifiers

		Training Performance			Cross validation		
			
Feature combination		true	false	ratio	true	false	ratio
Solv	Stab	20	4	83.33%	20	4	83.33%
Solv	Func	16	8	66.67%	16	8	66.67%
Solv	Clust	16	8	66.67%	16	8	66.67%
Stab	Func	23	1	95.83%	23	1	95.83%
Stab	Clust	20	4	83.33%	19	5	79.17%
Func	Clust	18	6	75.00%	17	7	70.83%

Abbreviations: Solv, Solvent Accessibility, Stab, Stability, Func, Functional Sites, Clust, Clustering

### Towards prediction

Given the good performance of the classifiers, we applied the classification to five genes with uncertain annotation (MMP2, PIK3C3, TGM3, EPHA3, DCLK3). These genes were not included in our original dataset either because they were not in the "Cancer Gene Census" category of COSMIC (MMP2, PIK3C3, TGM3, EPHA3) or because there was no crystal structure available (DCLK3). We generated a homology model for DCLK3 (Additional File 6, Figure S2). For EPHA3 we found clear evidence in the literature that it acts as a tumor suppressor [31]. For the other four genes, the classification is less clear. We systematically applied the linear classifiers shown in Figure 3 to this set. Table 3 shows a summary of the results. The consensus of the classifiers identifies DCLK3, MMP2, TGM3 as oncogenes and PIK3C3 and EPHA3 as tumor suppressors. This matches the prediction result of the best performing classifier (*functional sites *versus *stability*).

**Table 3:** Prediction of cancer gene classes

		Prediction				
		
Feature combination		DCLK3	MMP2	PIK3C3	TGM3	EPHA3
Solv	Stab	O	S	S	O	S
Solv	Func	S	O	S	O	S
Solv	Clust	O	O	S	O	S
Stab	Func	O	O	S	O	S
Stab	Clust	O	S	S	O	S
Func	Clust	S	O	S	S	S

Abbreviations: Solv, Solvent Accessibility, Stab, Stability, Func, Functional Sites, Clust, Clustering, O, Oncogene, S, Tumor Suppressor

## Discussion

Previous studies of structural effects of mutations have found that disease mutations primarily occur in the protein core [13,14]. We can confirm this trend only for the set of tumor suppressors. In contrast, core residues in oncogenes are significantly less often mutated than expected by chance. This is in agreement with our results for protein stability. Mutations located in the protein core are often destabilizing and result in loss-of-function. Thus, our data suggests that the loss-of-function of tumor suppressors is often caused by destabilization of the protein.

Similar to our findings, Gong and Blundell show that cancer mutations are less often located in solvent inaccessible areas than expected, as opposed to Mendelian disease-related variants [32]. In another recent study, Talavera et al. report that cancer driver mutations are more likely located on the surface of proteins than expected by chance [9]. Their observation that the patterns of cancer-associated mutations and common polymorphisms are "remarkably similar" can be explained by our results that the opposing trends of tumor suppressors and oncogenes neutralize each other when looking at cancer mutations in general.

Functional site mutations can either disable enzymatic activity and regulatory mechanisms or increase protein activity, as it has been described for several examples. One example is the well-characterized V600E mutation in BRAF that mimics the phosphorylation of the kinase domain activation segment [33]. For the *Onc *set we observed a significant overrepresentation of mutations proximal to functional sites. This suggests that specific mutations of functional sites are often responsible for oncogene activation. The underrepresentation of functional site mutations in the *Snp *dataset can be explained by the fact that SNPs are assumed to occur in the population without causing severe phenotypes. A mutation of a functional site impairing the native protein function would be unfavorable.

Our results show that the most frequently mutated types of functional sites in oncogenes are ATP and GTP binding sites and that the frequency of mutation is significantly higher than expected. This suggests that mutations of ATP and GTP binding sites are specific and common mechanisms of oncogene activation. In fact, examples for such activating mutations near ATP binding sites have been described in the literature [33-35]. This is supported by previous findings showing that the functional region of ATP binding is subject to a greater selection pressure indicative for the presence of candidate driver mutations [36], and that in kinases this site shows a higher proportion of driver mutations compared to the remaining catalytic domain [3]. Further, mutations in the GTP binding site of RAS genes have been described to impair GTPase activity. These mutations retain the protein in a GTP-bound state leading to constant activation of the gene [37,38].

We have observed highly significant spatial clustering of mutations in particular in oncogenes. Similar trends have been described in recent publications [39,40]. Even though different, sequence-based definitions of clustering were used, the results, like ours, support the hypothesis that mutations in specific regions in the structure are required for gene activation. Our results further indicate that tumor suppressor deactivation is a locally less constrained process.

To identify tumor-causing mechanisms from sequencing data it is important to distinguish between driver and passenger mutations. By definition, driver mutations are actively involved in the process of tumor formation. In contrast, passenger mutations occur by chance and do not confer any growth advantages. Typically, cancer genomics studies will include a step to filter out passenger mutations and several approaches for such filtering have been described [3,36,41,42]. We have only included genes that are taken from the "Cancer Gene Census" part of the COSMIC database and we make the assumption that mutations described in the literature are less likely to be passengers. Nevertheless, there is the possibility that the *Mut *dataset contains passenger mutations. We expect that they behave more similar to the control sets (*Rnd *and *Snp*) and shift the results towards the expected random value. Since the observed differences between *Onc *and *Sup *are so significant, we conclude that the signal from driver mutations dominates the noise induced by passengers.

Figure 3 shows the behavior of individual genes and the linear classifiers that we trained on the dataset. We find that plots with the *stability *feature on one axis (Figure 3A, 3D, 3E) show good separation. We looked at some outlier genes with unexpected behavior in more detail. For example, the value for functional site mutations in PIK3CA is zero. This is because the databases were missing annotations described in the literature for the ATP binding- and catalytic sites [43]. So there is some effect of database contents, but the other genes in our dataset seem to be well-annotated.

The two recurring outliers, PTPN11 and AKT1 are the genes with the least number of distinct mutations in our dataset. Therefore, we suggest that results for genes with few mutations should be handled with care and that for a robust classification more mutations are advantageous. Plots involving *clustering *(Figure 3C, 3E, 3F) show that all tumor suppressors have a similar clustering value around one, whereas oncogenes show a wider distribution with very high and some low values. The three members of the RAS family show the highest clustering values due to the dominance of mutations around the common hotspot at position twelve. KIT shows the lowest clustering value because it is only rarely mutated in the eight selected tumor types and the mutations are even more scattered in the structure than random.

The results of the cross validation showed good performance of the features for predictive classification. Hence, we used the classifiers to predict the functional class of five genes not included in the original dataset. We compared the predictions of our linear classifiers to recent results by Bozic et al. [44]. They conducted a classification of all genes contained in COSMIC into oncogenes and tumor suppressors based on non-structural features. For two of the genes (DCLK3 and MMP2) their classification as oncogenes matches ours. For EPHA3 the two annotations disagree. Our classification is in accordance with prior knowledge about the tumor suppressor activity of EPHA3 [31]. Further investigations may be required to elucidate this apparent disagreement. For two previously uncharacterized genes (PIK3C3 and TGM3), for which Bozic and coworkers do not report annotations, we suggest that they act as tumor suppressor and oncogene, respectively.

## Conclusions

The central contribution of this study is that it describes in a quantitative way, the opposing structural effects of cancer-associated missense mutations in oncogenes and tumor suppressors. With our findings we can confirm and statistically validate the hypotheses for the gain-of-function and loss-of-function mechanisms of oncogenes and tumor suppressors, respectively.

Moreover, we present a method that can be used to predict whether a newly identified gene likely acts as an oncogene or a tumor suppressor. The method uses structural features that, in lack of experimental structures, can be derived from predicted models. In our analysis we have focused on properties of cancer mutations that act at the structural level. Hence, our results give complementary information compared to methods that use sequence information alone. We have shown that our method performs well for predictive classification. This pre-classification of genes into functional classes will be a valuable tool in cancer research.

To further understand the complex mechanisms that lead to tumor initiation, the proteins have to be analyzed on a biochemical and functional level and in the context of their native interaction partners. Such investigations at the systems level are currently being performed for many of our target genes as part of the Mutanom project (http://www.mutanom.org). Ultimately, systems biology approaches that integrate genome-wide mutational and epigenetic analyses with structural and functional analyses as well as quantitative modeling of pathways will pave the way to predictive models of genetic diseases. Such prediction models will aid the development of individualized medicine approaches and diagnostics optimizing treatment efficiency and minimizing drug side effects.

## Competing interests

The authors declare that they have no competing interests.

## Authors' contributions

Conceived and designed the experiments: HS, SJJ, ML, BMHL, HL. Performed the experiments and analyzed the data: HS, SJJ. Contributed analysis tools and datasets and helped with data analysis: JMD, CW. Wrote the paper: HS, SJJ, BMHL. All authors read and approved the final manuscript.

## Supplementary Material

Additional File 1 Figure S1 - Overview of genes and mutations Click here for file

Additional File 2 **Table S1 - Genes**. Table columns: HUGO gene name, UniProt accession number, length in amino acids, cancer gene class Click here for file

Additional File 3 **Table S2 - Mutations**. Table columns: HUGO gene name, sequence position, wild type amino acid and mutant amino acid of the cancer-associated mutations used in the analysis, COSMIC ID, mutational information at the level of DNA Click here for file

Additional File 4 **Table S3 - SNPs**. Table columns: HUGO gene name, sequence position, wild type amino acid and mutant amino acid of the natural variants used in the analysis, reference SNP ID Click here for file

Additional File 5 **Table S4 - Annotations**. Table columns: HUGO gene name, sequence position, type of functional site, source database Click here for file

Additional File 6 Figure S2 - Structure model for DCLK3 Click here for file
