# Supplementary material for: The structural impact of cancer-associated missense mutations in oncogenes and tumor suppressors
Source: Mol Cancer. 2011 May 16;10:54. doi: 10.1186/1476-4598-10-54 (PMC3123651; doi:10.1186/1476-4598-10-54)
Supplement: Additional File 1 — Figure S1 - Overview of genes and mutations [file 1476-4598-10-54-S1.PDF]

Additional File 1 Figure S1

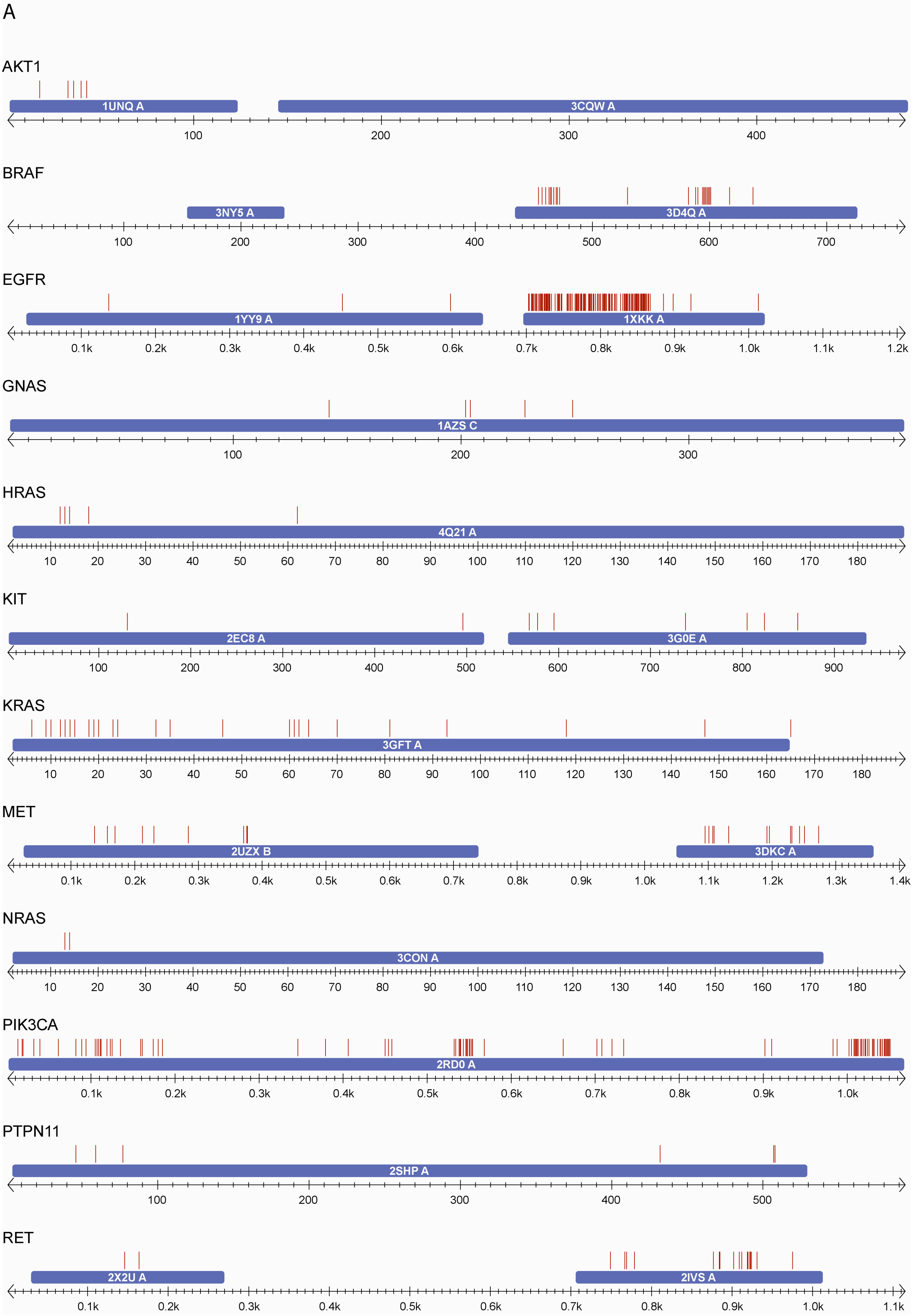

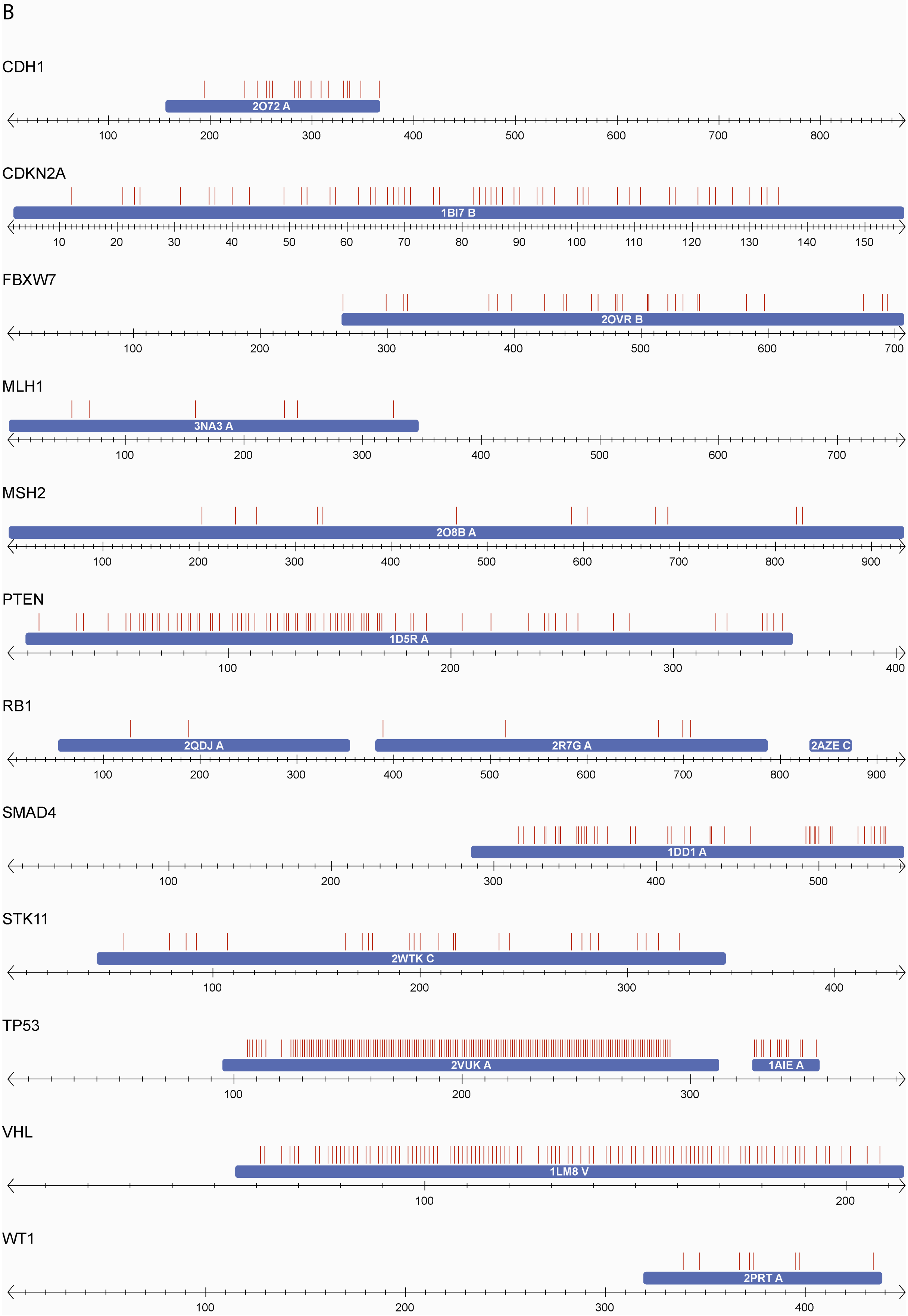
B
CDH1
2O72 A
100 200 300 400 500 600 700 800
CDKN2A
1BI7 B
10 20 30 40 50 60 70 80 90 100 110 120 130 140 150
FBXW7
2OVR B
100 200 300 400 500 600 700
MLH1
3NA3 A
100 200 300 400 500 600 700
MSH2
2O8B A
100 200 300 400 500 600 700 800 900
PTEN
1D5R A
100 200 300 400
RB1
2QDJ A
2R7G A
2AZE C
100 200 300 400 500 600 700 800 900
SMAD4
1DD1 A
100 200 300 400 500
STK11
2WTK C
100 200 300 400
TP53
2VUK A
1AIE A
100 200 300
VHL
1LM8 V
100 200
WT1
2PRT A
100 200 300 400

Additional File 1 Figure S1. Overview of genes and mutations. Regions with known crystal structures are shown as blue bars with the corresponding PDB code. Mutations are depicted as vertical red lines. A, all oncogenes used in the analysis. B, tumor suppressors used in this study.
