# Supplementary material for: The structural impact of cancer-associated missense mutations in oncogenes and tumor suppressors
Source: Mol Cancer. 2011 May 16;10:54. doi: 10.1186/1476-4598-10-54 (PMC3123651; doi:10.1186/1476-4598-10-54)
Supplement: Additional File 6 — Figure S2 - Structure model for DCLK3 [file 1476-4598-10-54-S6.PDF]

Additional File 6 Figure S2

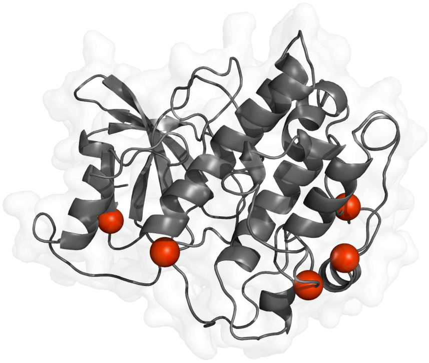

Additional File 6 Figure S2. Structure model for DCLK3. Cancer mutations are highlighted as red spheres. The homology model was built based on templates 2W4OA, 2JC6A and 1A06A. Templates were identified by a psi-blast search with 5 iterations. Suitable templates were manually selected based on sequence similarity, alignment quality and predicted secondary structure. Alignments were refined using a contact-based target to multiple template alignment. Finally, models were built using distance geometry and subsequent simulated annealing refinement.
